# *Acropora cervicornis* and *Acropora palmata* cultured on a low maintenance line nursery design in The Bahamas

**DOI:** 10.1371/journal.pone.0267034

**Published:** 2022-04-25

**Authors:** Leah Maurer, Lauren Puishys, Nancy Kim Pham Ho, Craig Dahlgren, Tanya Y. Kamerman, Scott Martin, M. Andrew Stamper

**Affiliations:** 1 Disney’s Animals, Science and Environment, Lake Buena Vista, Florida, United States of America; 2 New College of Florida, Sarasota, Florida, United States of America; 3 Perry Institute for Marine Science, Waitsfield, Vermont, United States of America; 4 Nova Southeastern University Oceanographic Center, Dania Beach, Florida, United States of America; University of Technology Sydney, AUSTRALIA

## Abstract

Acroporid corals are one of the most important corals in the Caribbean because of their role in building coral reefs. Unfortunately, *Acropora* corals have suffered a severe decline in the last 50 years thus prompting the development of many restoration practices, such as coral nurseries, to increase the abundance of these species. However, many coral nursery designs require constant visits and maintenance limiting restoration to more convenient sites. Additionally, most studies lack the details required for practitioners to make informed decisions about replicating nursery designs. Two line nurseries were monitored for three years in The Bahamas to assess the survival of corals, *Acropora cervicornis* and *Acropora palmata*, as well as evaluate the durability and cost effectiveness of the nursery design. Survivorship ranged from 70 to 97% with one location experiencing significantly higher survivorship. The initial year build-out cost was high for a nursery, $22.97 per coral, but each nursery was comprised of specific materials that could withstand high storm conditions. Some unique aspects of the design included the use of longline clips and large-diameter monofilament lines which allowed for easier adjustments and more vigorous cleaning. The design proved to be very durable with materials showing a life expectancy of five years or more. Additionally, the design was able to withstand multiple hurricanes and winter storm conditions with little to no damage. Only two maintenance visits a year were required reducing costs after construction. After three years, this nursery design showed promising durability of materials and survivorship of both *Acropora cervicornis* and *Acropora palmata* despite being serviced just twice a year.

## Introduction

Historically, acroporid corals dominated shallow reefs throughout the Caribbean region [1–4]. *Acropora cervicornis* and *Acropora palmata* were the structural base of reefs in the Caribbean and Florida Keys providing habitats and refuge for fisheries, assisting with nutrient recycling, and acting as coastal buffers [2, 5]. More importantly, these species are vital for reef development due to high growth rates and unique branching morphologies unlike any other corals in the region [2, 6, 7]. Unfortunately, *Acropora* has suffered an estimated population decline of approximately 95% in some areas since the 1970’s [3, 8–10] and were listed as critically endangered by the International Union for Conservation of Nature (IUCN) Red List of Threatened Species in 2008. Natural recovery has been limited due to changes to the physical environment, low recruit survivorship, and low rates of sexual recruitment as well as high variability in reproductive and settlement events [2, 9, 11–14].

Both species of *Acropora* are considered good candidates for active restoration due to their ability to reproduce through fragmentation, high growth rates and survivorship of fragments, and the ability to heal rapidly [7, 15–17]. Active restoration refers to projects that directly enhance coral abundance which can include strategies such as coral nurseries. There are multiple types of nursery designs: 1) floating line and Table 2) fixed bottom (block, frame or tree). Fixed nurseries attach coral to blocks (i.e cement or cinder) or metal/plastic frames anchored to the seafloor. These tend to be more durable in strong currents due to close proximity to the bottom but close proximity to the bottom also means higher risk of sediment/loose debris on the corals as well as biofouling agents. Additionally, fragments may have to be re-attached or readjusted as coral increase in size or get displaced by debris. Maintenance is required more frequently for fixed bottom designs in ensure coral success [18–21]. On the other hand, line nurseries suspend branching corals secured to hard structures (ie: polyvinyl chloride or fiberglass) through the use of monofilament/nylon, rubber coated wire, monel® wire, cable ties, plastic pins, and shielded wire, or by simply intertwining and securing the corals within braided lines at various depths [18, 22–29]. Corals on line nurseries have demonstrated high growth rates, low predation and macroalgae accumulation, and an ample supply of water flow and circulation for coral success [21, 26] making them very attractive for restoration. Additionally, line nurseries can be adjusted (length of lines, line height in water column or entire nursery) to optimize growth in response to storms or increased coral weight [18]. Nevertheless, every nursery type requires regular maintenance. Maintenance can include removal of fouling agents (algae, invertebrates and/or sediment), frequent separation of growing corals, and replacement of deteriorating materials [18]. When choosing a nursery design for a restoration project multiple parameters, such as available resources and labor, permitting regulations and the environmental conditions at the nursery site, need to be considered [19, 30].

The rapid expansion of coral nurseries in the Western Atlantic has led to numerous handbooks, manuals, and reviews of best practices [19, 21, 23, 30–39]. However, these resources omit key elements such as material specifications, labor hours and costs, which are critical to providing coral restoration practitioners the tools needed to make informed decisions [40]. Additionally, many published coral nursery studies are only short term projects [31] or have propagated coral successfully for many years, especially in Caribbean and Florida keys, but have not reported on it. Very few projects use both *A*. *cervicornis* and *A*. *palmata* on the same nursery [19, 31]. Furthermore, there is a need for more designs that require minimal visits and mechanical maintenance (replacement of nursery structure) for sites that are harder to access. Line nurseries, which are considered a low maintenance design [21], can be beneficial for small teams accessing remote locations that limit visitation. Unfortunately, line nurseries, which utilize more of the water column compared to fixed bottom nurseries, are subjected to more wave action generated by storms [18] thus materials must be carefully selected to withstand strenuous environmental conditions, especially since the number of tropical storms and hurricanes have continued to increase over the last century [41]. More durable low-maintenance and cost-efficient line nursery designs are required to support successful active coral restoration especially in remote storm-prone areas. In this study, we report the survival of *A*. *cervicornis* and *A*. *palmata* as well as the durability and cost effectiveness of a line nursery design in a remote storm-prone area of The Bahamas.

## Methods

### Location of nurseries

**Fig 1:**
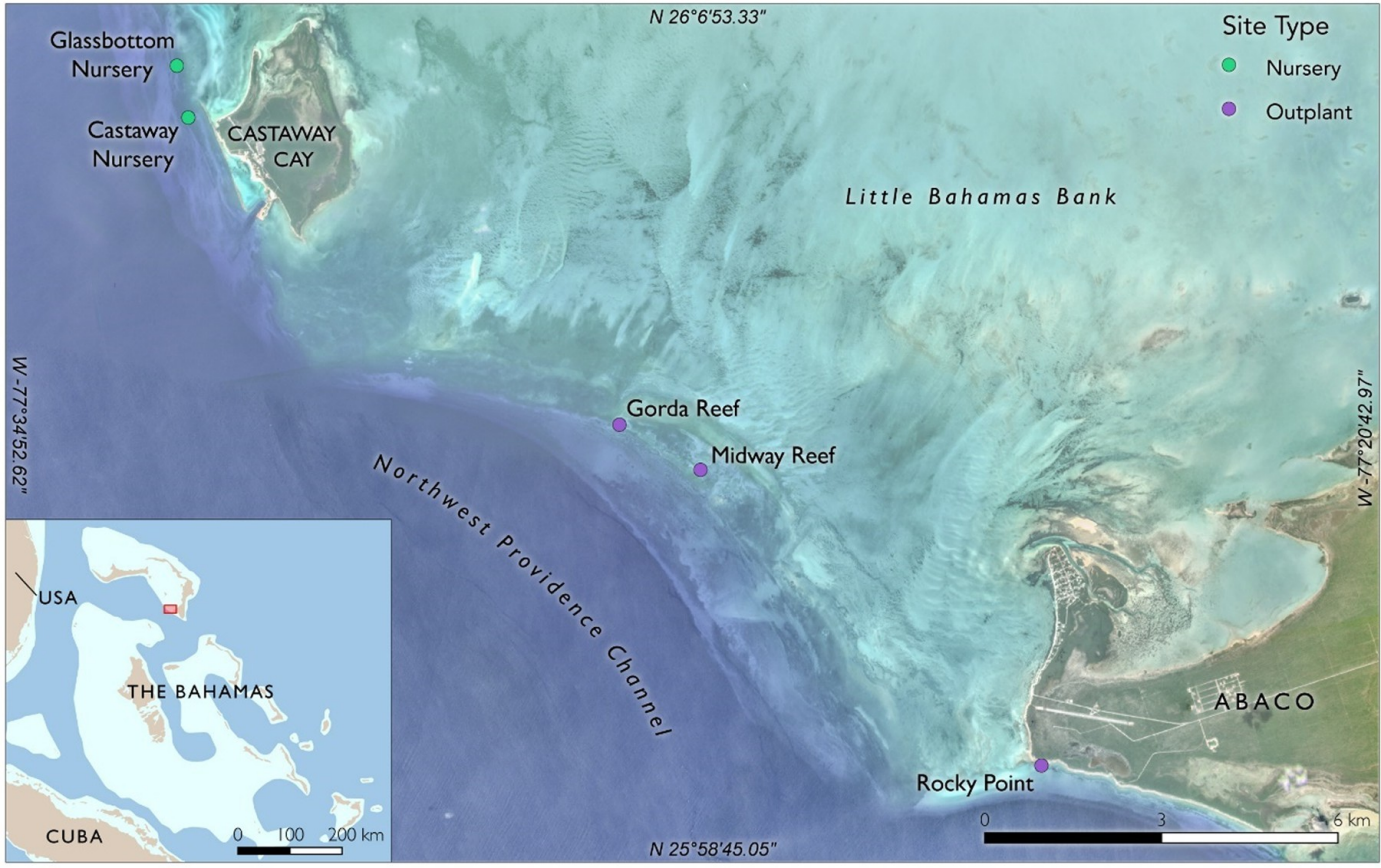
Location of two coral nurseries near Disney Castaway Cay, The Bahamas made using the free and open source geographic information system QGIS. Nursery locations are indicated by green dots.

Corals were grown in two line nurseries along the margin of the Little Bahama Bank 12–15 m deep off Gorda Cay, Abaco, The Bahamas: Castaway (26°05’38.0"N 77°32’59.5"W) and Glassbottom (26°06’06.5"N 77°33’06.8"W) (Fig 1). Castaway is approximately 500 m west of Castaway Cay and Glassbottom is approximately 1 km to the north of Castaway Cay, Disney’s private cruise line island port. Both nurseries are set in line with a spur and groove calcium carbonate hard bottom. Local marine mammal experts were consulted to assure the nurseries were not located in high marine mammal traffic areas. This project was conducted under permit MAMR/FIS/17 of the Bahamian Department of Marine Resources.

### Construction of nurseries

All specific material and costing details are available in the S1 and S2 Tables. S1 and S3 Figs visually depict the materials used for each part of the nursery design.

**Fig 2:**
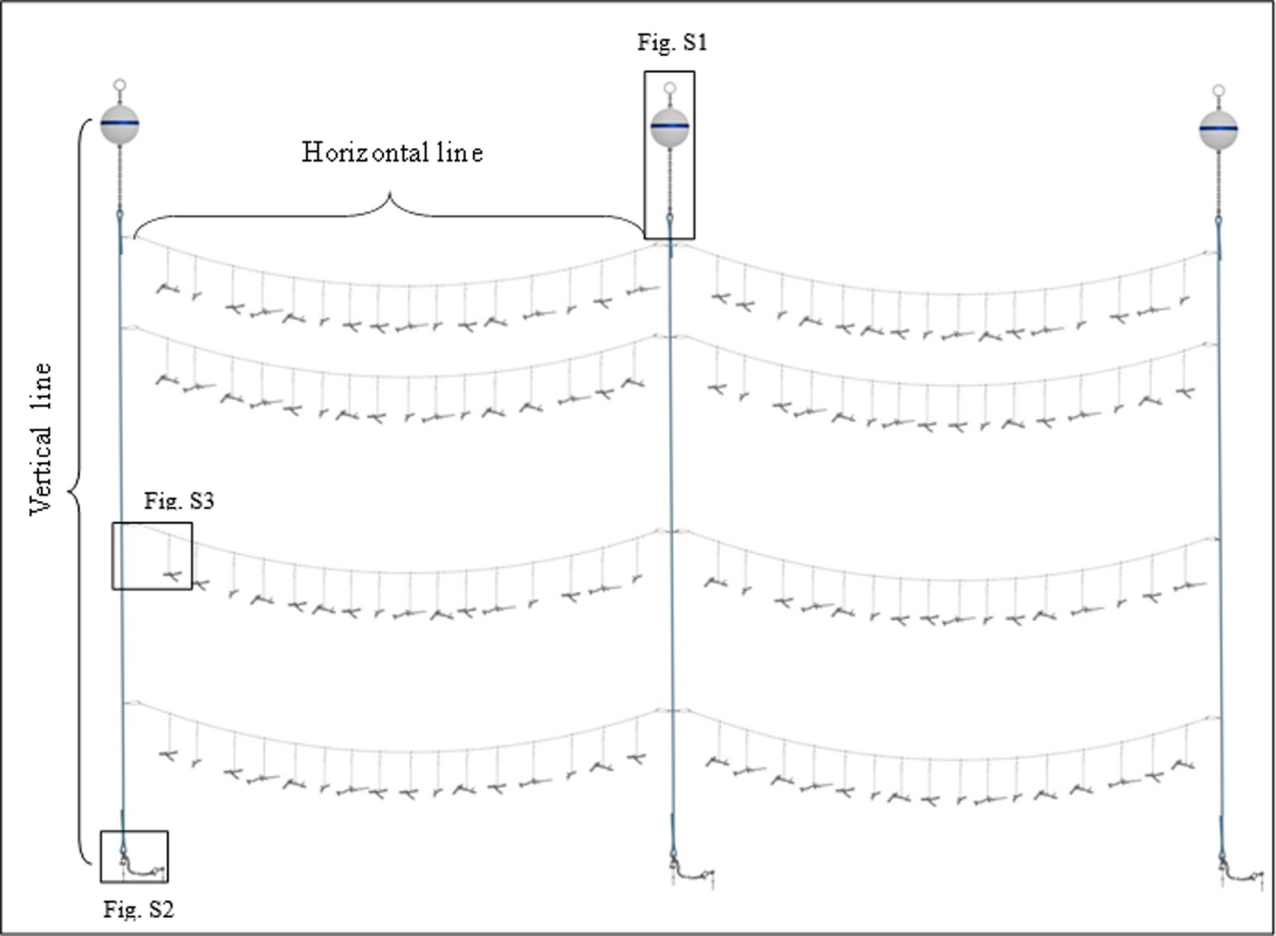
Diagram of line nursery built using three vertical lines and eight horizontal lines. Each nursery supports 128 coral individuals with a footprint of approximately 8.2 meters tall and 14 meters wide.

Each nursery utilized three vertical mooring lines made of 0.5 inch three stranded Samson rope suspended in the water column using a subsurface closed cell foam buoy and eight horizontal monofilament (2.8 mm diameter) lines strung between moorings, from which 128 coral pieces were suspended (Fig 2). Nurseries were positioned such that horizontal lines ran perpendicular to the prevailing tidal currents, allowing suspended corals to swing freely and reduce entanglement. The total height of the vertical line, including all the connecting hardware, was approximately 8.2 m. The buoy remained 2–3 m below the water surface to minimize wave action and to prevent boat strikes (S1 Fig). Each vertical line was anchored 7 m apart into the hard bottom. A secondary anchor was placed about 0.6 m from the original anchor in case of detachment and as an extra precaution for each vertical line (S2 Fig).

Horizontal lines ran parallel to one another and were spaced along the vertical line at depths of approximately 6 m, 7.5 m, 9 m, and 10.5 m from the surface. The horizontal lines were secured by looping nylon and polypropylene mix line to the vertical lines. Stainless steel longline clips, with the swivel attachment removed, connected the nylon and polypropylene mix loop to the 7 m long horizontal lines (S3 Fig). Each of the eight horizontal lines supported 16 coral pieces spaced approximately 30 cm apart to allow for growth and prevent entanglement. The corals were suspended onto the horizontal lines using a stainless steel longline clip to allow movement in currents. Double barrel crimps were placed on either side of each coral to prevent sliding along the horizontal line (S3 Fig).

Cost of equipment and consumables were reported in USD ($) and labor was reported in hours for each project phase following Edwards et al. [35]. The phases of the project consisted of: (1) nursery construction, and (2) nursery maintenance. Labor hours were logged by individual for each task and represent time taken to complete the activity. The number of boat days and SCUBA tanks needed per phase was also noted. The cost per coral was calculated by totaling the cost of equipment needed to construct the nursery, nursery materials, and nursery maintenance materials, and dividing by the number of corals supported by one nursery at initial start-up (128) (S2 Table).

### Environmental monitoring

Temperature (°C) and light intensity (lux) were measured using HOBO Pendant® Temperature/Light 64K data loggers (ONSET Computer Corporation, Bourne, MA). We deployed one HOBO logger to the top (shallow) and bottom (deep) horizontal lines of both nurseries via heavy duty cable ties. The HOBO loggers were programmed to record temperature and light intensity in 20 or 30 minute intervals. At each trip to the nurseries, the HOBO loggers were removed, data was downloaded for analysis using a HOBO® Waterproof Shuttle and HOBOware software, and a new logger was installed. Due to algal fouling on the loggers, the time period for light comparison was limited to the first seven days after each redeployment.

### Nursery corals

To stock the nursery, donor *A*. *cervicornis* and *A*. *palmata* were harvested from sites within 20 km of each nursery site in May 2016. Healthy fragments were removed from adult donor corals from nearby wild reefs using bolt cutters or collected from naturally fractured pieces found at the bottom. Each *A*. *palmata* fragment was cut into approximately 5 x 5 cm pieces and each *A*. *cervicornis* fragment was cut into approximately 5 cm long pieces with a wet tile saw and attached to a monofilament line. Fragments remained in seawater, except during the cutting process. Castaway and Glassbottom nurseries were each stocked with 32 *A*. *cervicornis* and 96 *A*. *palmata* fragments (Table 1). A small sample of each donor was preserved in about one milliliter of molecular grade ethanol for genetic analysis to determine if donor colonies represented unique genotypes. DNA was extracted using magnetic bead protocol [42] followed by PCR amplification using five microsatellite markers [42–45]. GeneMapper 5™ software was used to determine peaks for each fragment loci and genotypes were confirmed with matching loci using the Excel microsatellite toolkit [45]. Donor colonies were randomized when placed on the nurseries using a random number generator. Through genetic analysis, we identified only one genotype of *A*. *cervicornis* among the five colonies tested, with one inconclusive test. However, we identified four distinct *A*. *palmata* genotypic groups which we named after their collection reef; Rocky (three donor colonies), Rocky 2 (one donor colony), Gorda (one donor colony), and Midway (three donor colonies). Data for two of the *A*. *palmata* colonies came back inconclusive, both of which were only represented at Glassbottom.

**Table 1:** Number of corals from different colonies for two coral lines nurseries at varying depths in The Bahamas.

Nursery	Depth (m)	Species	Donor Colony	# of Individuals
**Castaway**	10.5	*A*. *cervicornis*	ACER1	9
		*A*. *palmata*	Gorda	4
			Midway	8
			Rocky	8
	9		Rocky2	4
		*A*. *cervicornis* *A*. *palmata*	ACER1	8
			Gorda	4
			Midway	8
			Rocky	8
	7.5		Rocky2	4
		*A*. *cervicornis* *A*. *palmata*	ACER1	7
			Gorda	4
			Midway	8
			Rocky	8
			Rocky2	4
	6	*A*. *cervicornis*	ACER1	8
		*A*. *palmata*	Gorda	4
			Midway	8
			Rocky	8
			Rocky2	4
**Glassbottom**	10.5	*A*. *cervicornis*	ACER1	8
			Unknown	4
		*A*. *palmata*	Midway	8
			Rocky	4
			Unknown	8
	9	*A*. *cervicornis*	ACER1	8
			Unknown	4
		*A*. *palmata*	Midway	8
			Rocky	4
			Unknown	8
	7.5	*A*. *cervicornis*	ACER1	8
			Unknown	4
		*A*. *palmata*	Midway	8
			Rocky	4
			Unknown	8
	6	*A*. *cervicornis*	ACER1	8
			Unknown	4
		*A*. *palmata*	Midway	8
			Rocky	4
			Unknown	8

It was determined that all of the *Acropora cervicornis* was a clone so it was designated as ACER1. Each *A*. *palmata* colony was named for the reef it was retrieved from.

During the first of the two annual nursery inspections, corals were harvested for outplanting. Harvest included cutting new growth from individuals on the nursery. At the end of year one (June 2017), four coral fragments from every source colony of each species were haphazardly harvested from Glassbottom. No corals were harvested at the Castaway. At the end of year two (June 2018) and three (June 2019), corals that displayed linear growth larger than 15 cm were trimmed for outplanting for both nurseries to ensure outplant survival and growth [26].

### Maintenance and monitoring

The nursery was designed to require minimal and infrequent maintenance. Both nurseries were maintained every six months. For each nursery, five divers would visually inspect nursery integrity, conduct necessary repairs, and remove biofouling such as algae and encrusting fire coral (*Millepora alcicornis*) from the lines. Survival was monitored at every nursery visit by recording the status of each coral as dead or alive.

### Statistical analysis

Temperature and light, as well as survival were compared between nurseries and line depths using non-parametric statistical analyses (Wilcoxon rank sum tests and Kruskal-Wallis one way analyses of variance) due to non-normally distributed data. Binomial Generalized Linear Models were performed comparing the relationship between survival and depth as well as survival and genotype. Statistical analyses were performed using RStudio 3.6.1 [46].

## Results

### Construction

The custom-designed line nurseries described in this study required only two visits a year. With five divers, two hours were required to visually inspect each nursery, conduct necessary repairs, and remove bio fouling such as algae and branching fire coral (*Millepora alcicornis*) from the lines. This resulted in ten labor hours to maintain 128 corals (4.7 minutes per coral) for each visit after construction. Additionally, our line nurseries have been exposed to hurricanes and tropical storm conditions from Hurricane Dorian, Irma, and Maria and Tropical Storm Philippe [47–50] and experienced minimal damage as a result of these events. Furthermore, outside of hurricane season, winter cold fronts can produce sustained winds of tropical storm or hurricane force one or more times each year (C. Dahlgren, pers. obs.). One weak point was detected in our design over the course of three years. Twice a coral was found missing due to a failure in the swivel of the longline clips (S3F Fig).

Our current design has an initial build-out and labor cost of $2,940.52 (items priced for 2018) (S1 and S2 Tables). Our initial cost per coral housed on the nursery is $22.97 (USD). Additionally, we were able to construct one nursery with six divers over two days with a total of 18 labor hours (S2 Table). For the first year, labor would cost $1,409.96 US (minimal wage of $8.65 in 2018) but for subsequent years, labor would only cost $865 US to complete the two maintenance visits. To date, the horizontal lines for our nursery design require replacement after five years, and the vertical lines and buoys have yet to be replaced after seven years.

### Environmental parameters

Environmental parameters by nursery and line are summarized in Table 2. Mean temperatures at Castaway were significantly higher than those at Glassbottom (Wilcox Test, w = 1.167e+10, *p* = 2.2e-16). However, Castaway and Glassbottom had an overall mean difference of 0.1°C which may not be biologically significant. The difference in mean temperatures was within the margin of error of the instrument used (HOBO Pendant® Temperature/Light 64K Data Logger) and the statistical difference was most likely caused by high levels of replicate observations from loggers producing a data point every 20–30 minutes. Additionally, Castaway had significantly lower levels of light, with corals on Castaway’s shallowest line experiencing just 17% more light (in lux) than Glassbottom’s deepest line (Kruskal-Wallis Test, X^2^ = 122.74, *p* = 2.2e-16, *df* = 3). Both nurseries experienced about 60% transmission loss in light from top to bottom line. Light values converged at zero during the overnight hours, and experienced their peak differences towards midday. We excluded light data for fall 2018 as one of the data loggers was compromised.

**Table 2:** Summary of environmental parameters (± SE) monitored at two coral line nurseries in The Bahamas.

		Temperature (°C)		Light (lux)	
		Castaway	Glassbottom	Castaway	Glassbottom
**Mean**	Overall	27.5 ± 0.006	27.4 ± 0.005	3215 ± 119	4344 ± 154
	Top Line	27.6 ± 0.009	27.4 ± 0.008	3961 ± 194	5361 ± 260
	Bottom Line	27.3 ± 0.008	27.4 ± 0.008	2470 ± 135	3367 ± 164

### Coral survival

Year had a significant effect on survival (Kruskal Wallis, X^2^ = 14.83, *df* = 2, *p* = 6.021e-04). Overall survival (of both species at both nurseries) decreased 8% between year one and year two, dropping from 96% to 88%. Overall survival was 86% by year three, which was not significantly lower than the previous year. *A*. *palmata* survival at Castaway drove this effect as it was the only treatment group to see a significant drop between years one and two (Kruskal-Wallis X^2^ = 13.918, *df* = 2, *p* = 9.499e-04). Broken down by nursery, Glassbottom had 96% survival, significantly higher than the 74% survival at Castaway (Wilcox Test, w = 5841.5, *p* = 1.128e-06). When additionally analyzed by species, both *A*. *cervicornis* and *A*. *palmata* individually saw higher survival rates at Glassbottom (Wilcox Tests: *p <* 0.05). At Glassbottom, there was a 94% and 97% survival rate of *A*. *cervicornis* and *A*. *palmata* respectively, and only a 70% and 75% survival rate of *A*. *cervicornis* and *A*. *palmata* respectively at Castaway.

Both harvest method and depth showed no significant effect on survival. Harvesting corals during year one only, year two only or during both years had no significant effect on survival (Kruskal Wallis, X^2^ = 0.47, *df* = 2, *p* = 0.79). Even though depth was not a significant predictor of coral survival (Binomial GLM, *p* > 0.05), we observed an interesting pattern, with *A*. *palmata* and *A*. *cervicornis* showing opposite trends. *A*. *cervicornis* had the highest survival on the center two lines, whereas *A*. *palmata* had the highest survival on the top and bottom lines.

**Fig 3:**
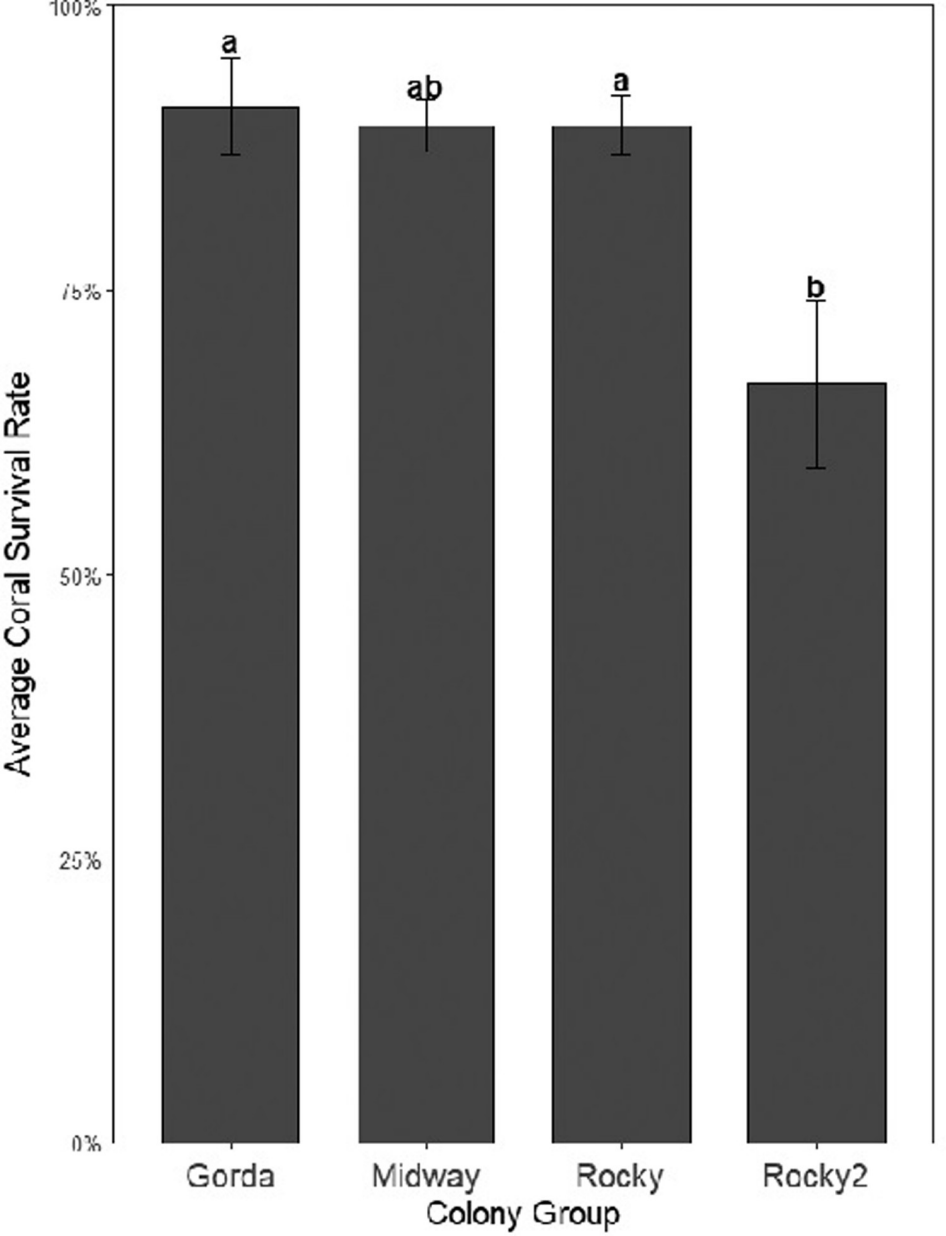
Average (±SE) coral survival rate (%) of four *Acropora palmata* donor colonies grown on horizontal line nurseries, Abaco, Bahamas after three years. Letters group the average coral survival rate in which statistically significant differences occurred (*p* < 0.05).

*A*. *palmata* had multiple genotypes identified, however, only two of these genotypes (Rocky and Midway) were included on both Castaway and Glassbottom. When only analyzing those genotypes present on both nurseries, Glassbottom still showed higher overall survival compared to Castaway (Wilcox Test, w = 1246, *p* = 0.02393). Of the two genotypes represented on both nurseries, only Midway saw significantly higher survival at Glassbottom (Wilcox Tests: Midway, w = 3912, *p* = 5.904e-03; Rocky, w = 2038, *p* = 0.0781). Genotype had a significant effect on survival (Binomial GLM, *p* < 0.05). Of the four unique genotypes of *A*. *palmata* in the nursery system, Rocky2 had a significantly lower survival rate than Rocky and Gorda, while Midway fell in the middle of the groups (Kruskal Wallis, X^2^ = 10.694, *df* = 3, *p* = 0.0135, Fig 3).

## Discussion

Rearing coral on a nursery is an increasingly common method for restoring populations of Acroporid corals in the Caribbean [25, 27, 32]. However, many handbooks, manuals, and reviews of best practices [19, 21, 23, 30–39] do not go into specifics of constructing a nursery, mainly due to the fact that designs are tailored to local conditions and availability of materials can be uncertain. Furthermore, it is often not wise to replicate other designs unless site specifications are similar but we believe our nursery design can be successful for projects utilizing a line nursery specifically in remote storm prone locations. We demonstrate that our line nursery design can be maintained with only two site visits per year and can successfully propagate both *A*. *cervicornis* and *A*. *palmata* on the same nursery in an area frequently subjected to tropical storms and hurricanes.

Some unique aspects of our design in the application of long line clips and larger diameter monofilament lines. The long line clips allowed for easy replacement if a coral was lost or died and also allowed the coral to move with the currents without harming nearby corals. Furthermore, our use of large-diameter sized monofilament permitted rigorous removal of biofouling without compromising the lines thus reducing the need for more visits. Since the deployment of our nursery design in 2016, no structural damage has occurred to the vertical and horizontal lines. During this time the nursery has experienced hurricane and tropical storm conditions from Hurricane Dorian, Irma and Maria and Tropical Storm Philippe [42–45]. Outside of hurricane season, local residents reported hurricane strength winds and wave action and numerous winter storms, but the exact weather conditions the nursery experienced could not be determined. We can infer that our design is suitable for withstanding strong storms, but more data is needed to determine the exact weather conditions at the study site.

A downside to line nurseries is the possibility of animal entanglement [21]. Therefore, to minimize the probability of entanglement, the tension on each horizontal line was increased which also reduced sagging from the weight of growing corals and fouling organisms. No animals have been found entangled since deployment of our nursery design. Only two out of 128 corals were physically lost from the structure over the entire three-year period due to the swivels of the longline clips (S3F Fig). Even though the swivels allowed the suspended coral to freely spin with the currents, we suggest removing the swivels for future projects to ensure greater success.

Survivorship of acroporids on nurseries in the Caribbean has been reported [19, 29, 46], yet publications describing costs and labor remain scarce. One purpose of our study was to elaborate the costs associated with deploying and maintaining a successful line nursery in a remote location. However, it is important to note that making a direct cost comparison can be difficult given the cost of certain facets varies by project goal, location and time (e.g. necessary travel accommodations, staff wages, SCUBA gear costs, and local duty taxes on materials and permits). One way to decrease labor costs is utilizing local volunteers especially for simple maintenance visits following construction. Other nursery designs, such as mid-water floating ropes, trees, tables, or fixed-to-bottom trays, can reduce initial costs but their build-out materials are strategically selected to be economically feasible and low-tech for their site location, and require a more frequent maintenance regime [20, 23, 28, 51–53]. Although our initial cost per coral housed on the nursery is high at $22.97 (USD) compared to similar designs in remote areas [54, 55], our build-out materials were selected specifically to withstand the conditions of the environment for an extended period of years without requiring replacement, thus decreasing maintenance and laborers compared to other designs [20, 23, 28, 52, 53]. To date, the horizontal lines for our nursery design require replacement after five years, and the vertical lines and buoys have yet to be replaced after seven years. However, given the remoteness of the site and the importance of the horizontal lines, it is recommended to proactively replace the horizontal lines as deemed fit.

Labor hours are also difficult to compare as they are dependent on scale, goal of the project, and skill level of staff. However the available time and labor allocated for a project can heavily influence which design practitioners are able to use therefore we provided the labor time required to construct and maintain our nursery design (S2 Table). Since our materials did not require replacement with each visit, most of our labor time consisted of cleaning fouling organisms off the nursery lines. Fouling is very site specific and depends on the presence of fouling organisms and species that may predate them so labor may vary in other locations. Our nurseries required a total of 20 hours of maintenance with five divers and two site visits a year (S2 Table). Besides removal of fouling agents and replacement of deteriorating material, nursery maintenance can also include frequent separation of growing corals [18]. Our nursery design employed crimps to prevent the corals from moving along the horizontal lines (S3D Fig) eliminating the concern of fusion between fragments or harmful interactions between different species which can be an issue for other nursery designs [18]. Our application of the crimps on the horizontal lines removed any need to adjust corals thus decreasing maintenance during the visits.

In addition to demonstrating that our line nursery design is low maintenance, our design showed successful propagation of both *A*. *cervicornis* and *A*. *palmata* on the same nursery. Several studies have reported success of acroporids on nurseries, however, few publications follow both *A*. *cervicornis* and *A*. *palmata* grown on nurseries [19, 31]. Survivorship ranged from 70 to 97% with both species reared on a nursery. We did see a decrease in survival over time given the fact some individuals were lost possibly due to structural issues, specifically the fragility in the swivels of the stainless-steel longline clips, as well as mortality from bleaching. The exact source of mortality was often difficult to determine. However, one explanation for mortality is coral abrasion from the lines, particularly on the top lines which are expected to have the highest wave energy.

There was also differences in survival between the two locations despite the close proximity. Glassbottom experienced the highest survival rate of about 96% whereas Castaway experienced a survival rate of about 73%. Differences in survival could be attributed to differences in environmental conditions. Although depths were similar at both nurseries, with a maximum depth of 12–15 m at both locations, Castaway experienced significantly lower levels of light compared to Glassbottom, possibly influencing survival of the coral. Based upon our observations, Castaway experienced higher turbidity. Upon visits to the nurseries, Glassbottom could always be clearly seen from the surface, whereas Castaway could only be seen while underwater. Better water clarity at Glassbottom would allow more light to penetrate deeper into the water column, thus reaching the deeper coral individuals.

Despite being statistically significant, temperature differences at the two nurseries may not be ecologically significant since the difference in mean temperatures was within the margin of error of the instrument used (HOBO Pendant® Temperature/Light 64K Data Logger). However, temperature is a well-known factor that influences coral. It has been recorded that a rise of 0.1°C in sea temperatures can trigger bleaching events and differences in survival rate among locations can be induced by differences in water temperatures [56, 57]. No other environmental parameters were monitored, however better survival rates at one nursery may be attributed to increased water circulation, minimal sedimentation, and a reduction in predation and disease [36]. Small differences in the environment can have significant impacts on production of corals on nurseries.

In addition to abiotic factors such as temperature, coral genetics can also play a role in survival [51, 58, 59]. Genetic diversity serves as a way for populations to adapt to changing environments. With more variation, it is more likely that some individuals in a population will survive and acclimate to changing environmental conditions. Shearer et al. [60] suggest that coral restoration projects should contain 10–35 randomly selected local donor colonies to retain at least 50–90% of the genetic diversity of the original population. In this study, however, natural populations of *Acropora* species had been greatly reduced. All locations with *Acropora* within 20 km of the nurseries were sampled for donor colonies and there was only one locally available genotype for A. cervicornis. Acropora palmata diversity was limited by the nursery capacity and replication needed for comparisons among depths and nurseries.

A nursery’s durability, in regard to both normal marine wear and tear and storm surge, is essential for remote areas prone to extreme weather such as The Bahamas. The tradeoff to the current study’s initial high cost is that our design uses higher grade materials which hold well against storms and do not require frequent maintenance or visits. After three years, this design has shown promising durability of materials and survivorship of both *A*. *palmata* and *A*. *cervicornis*. Therefore we think this nursery design is worth pursuing in other remote locations.

## Supporting information

S1 Table Detailed description of each item required for nursery construction and maintenance including manufacturer and cost at year 2018. (PDF)

S2 Table Overview of monetary costs and labor hours required to build and maintain line nursery. Peripheral activities, such as travel time to and from sites and preparation of activities, are not included. Costs are in USD ($).
(PDF)

S1 Fig Diagram of the top of the vertical line. There are three vertical lines at which the top consisted of: (A) o-ring (B) 8 mm (5/16) anchor shackle (C) buoy collars (D) mooring buoy (E) steel chain (F) thimble (G) custom-ordered vertical line.
(TIF)

S2 Fig Diagram of the vertical line anchor point. (A) custom-ordered vertical line (B) thimble (C) 13 mm (½ in) anchor shackle (D) cable ties (E) 8 mm (5/16 in) anchor shackle (F) eyebolt (G) nut and washer (H) 13 mm (1/2 in) steel chain (I) second eyebolt set.
(TIF)

S3 Fig Diagram of the horizontal line secured to the vertical line. (A) custom-ordered vertical line (B) Two loops of 0.47 cm (3/16 in) diameter nylon line (C) 12.7 cm (5 in) stainless steel long line clip (D) 3.0 mm double barrel crimp (E) 2.8 mm diameter monofilament (F) 7.62 cm (3 in) long line clip (G) 2.0 mm double barrel crimps (H) 1.8 mm diameter monofilament line and (I) coral input.
(TIF)

S1 Data (XLSX)
