# Supplementary material for: Acropora cervicornis and Acropora palmata cultured on a low maintenance line nursery design in The Bahamas
Source: PLoS One. 2022 Apr 25;17(4):e0267034. doi: 10.1371/journal.pone.0267034 (PMC9037939; doi:10.1371/journal.pone.0267034)
Supplement: S1 Table — (PDF) [file pone.0267034.s001.pdf]

Phase	Item	Description		Item Specifications	Purpose of item	Manufacturer	Amount sold	2018 cost	cost/unit (US$)	# units needed for 1 nursery	Cost ($)	Subtotals	Grand Total
**Construction of Nursery**	**Specialty items**	**Metric Description**	**Standard Description**										
	Custom ordered vertical Line	1.27 cm diameter, 6.1 m long	1/2 in	Samson rope ultra blue three-strand line (strand bi-polymer olefin fiber. Cover fiber: bi-polymer, core fiber: olefin, working load limit (6,100 lb.) Custom spliced for 1.27 cm (1/2 in) thimble rigging at each end of line.	vertical Line anchored to sea floor	John Sakash Company	each	$95.00	$95.00	3	$285.00		
	Mooring Buoy closed-cell foam protected by a polyethylene shell	38.1 cm diameter with 50.8 mm tube diameter	15 in diameter with 2" tube diameter	27.2kg (60 lb.) buoyancy	lift vertical line	Taylor Made Products	1	$117.99	$117.99	3	$353.97		
	Underwater Two-Part Epoxy Putty	1.89 L	1/2 gallon	Z-Spar Splash Zone A-788 Kit	reinforces bolt into ground	Pettit Paint	each	$97.74	$97.42	1	$97.74	$736.71	
	**Hardware**												
	O-ring	10.16 cm inner diameter , 1.24 cm thick	4 in inner diameter, 1/2" thick	hot dipped galvanized	hold buoy in desired location	Sea-Dog Line	each	$7.99	7.99	3	$23.97		
	Buoy Collar	50.8mm diameter x 50.8mm depth	2 in diameter x 2 in depth	hot dipped galvanized steel	protects buoy from anchor chain wear, prolonging the life of the buoy	Taylor Made Products	each	$24.99	$24.99	6	$149.94		
	Galvanized Anchor shackle	8 mm	5/16 in	working load limit 681 kg (1,500 lb.)	attach ring to 3/8" chain	Columbus McKinnon Corp	each	$8.31	$8.31	9	$74.79		
	Galvanized Anchor shackle	13 mm	1/2 in	working load limit 1,816 kg (4,000 lb.)	connects vertical line to chain in buoy	West Marine	each	$9.29	$9.29	9	$83.61		
	1.2 m (4 ft.) length 9.5 mm (3/8 in) steel chain	9.5 mm	3/8 in	tangle-resistant grade 30 galvanized steel chain, working load limit 1,202 kg (2,600 lb.)	chain runs through buoy	Peerless Chain Company	per foot	$7.93	$7.93	12	$95.16		
	61 cm (2 ft.) length 13 mm (1/2 in) steel chain	13 mm	1/2 in	tangle-resistant grade 30 galvanized working load limit	base chain	Peerless Chain Company	per foot	$12.99	$12.99	6	$77.94		

			2,041 kg (4,500 lb.)								
Eyebolt with nut	20.32 cm long, 9.5mm diameter hole	8 in long, 3/8 in diameter hole	coarse stainless steel	anchor for vertical line	Lehigh	each	$3.68	$3.68	6	$22.08	
Washer	9.5mm x 3.81 cm	3/8 in x 1-1/2	zinc-plated steel fender	helps hold eyebolt in place	Crown Bolt	100	$20.37	$0.20	6	$1.22	
Crimp sleeves (2.0 mm)	N/A	N/A	aluminum double barrel crimp sleeves 8 Shaped	secure coral to 1.8 monofilament and clip	Catch all Tackle	1 bag =500	$25.99	$0.05	256	$13.31	
Longline clips	7.62 cm	3 in	stainless steel	attach coral fragments to horizontal line	Catch all Tackle	1 bag = 50	$38.99	$0.78	128	$99.81	
Longline clips	10.2 cm	4 in	stainless steel	attach horizontal line to vertical line	Catch all Tackle	1 bag = 50	$54.99	$1.10	16	$17.60	
Crimp Sleeve Double Barrel (3.3 mm)	Inner diameter 3.175 mm	Inner diameter 1/8 in	aluminum Double barrel Crimp Sleeves 8 Shaped	keeps long line clips in place on 3.0 mm monofilament line	Lindgren Pitman	1 bag = 500 pieces	$62.50	$0.13	272	$34.00	$693.43
**Monofilament/Plastic**											
128 strips of 35 cm (14 in) 1.8mm monofilament line	1.8 mm	0.0708 in	136 kg (300 lb.) test	suspends coral onto clip	Lindgren Pitman	by mass (5lbs)~2,650 ft.	$43.75	$0.02	1792	$29.58	
8 strips of approximately 10 m 3.0 mm monofilament line	2.8 mm	0.11 in	294 kg (650 lb.) test	horizontal lines	Lindgren Pitman	by mass (5lbs)~1,105 ft.	$43.75	$0.04	180	$7.13	
16 strips of 30.5 cm (12 in) nylon line	0.47 cm diameter	3/16 in diameter	white diamond braid nylon (nylon and polypropylene mix). Working load limit 40.8 kg (90 lb.)	attaches to vertical line to insert 4" longline clip	Everbilt	roll = 50ft	$8.21	$0.16	16	$2.63	
Cable ties	20.32 cm	8 in	UV resistant double locking black , 34 kg (75 lb.) test	back up reinforcement	Commercial Electric	pack=100	$7.32	$0.07	40	$2.93	$42.27
**Tools**											
Air drill	1.27 cm	1/2 in	reversible air drill	make hole for eyebolt anchor	Central Pneumatic	each	$24.99	$24.99	1	$24.99	
Round-shank masonry bit		1-1/2 in size, 12 in overall length		fitting for drill	B & A Mfg. Company	each	$129.96	$129.96	1	$129.96	
Air drill accessory kit				for drilling	Central Pneumatic	each	$8.99	$8.99	1	$8.99	
Garden Hoe				removes sand for drilling	Ames	each	$16.98	$16.98	1	$16.98	

	Small crimper			handles sleeves from 0.1 mm - 2.2 mm	crimps	American Fishing Wire	each	$27.99	$27.99	2	$55.98		
	Offshore large crimper			handles sleeves from 0.8mm - 3.3mm	crimps	American Fishing Wire	each	$139.99	$139.99	1	$139.99		
	Screw-On Clamp Fittings	1.59 cm-1.91cm ID hose	5/8 in-3/4 in ID hose	nylon male and female coupling	connects air drill to scuba tank	Fiskars Brands Inc.	each	$1.80	$1.80	2	$3.60		
	Screw-On Clamp Fittings	1.59 cm-1.91cm ID hose	5/8 in-3/4 in ID hose	nylon male and female coupling	connects air drill to scuba tank	Fiskars Brands Inc.	each	$2.00	$2.00	2	$4.00		
	Hose	15.24 meters, 1.59 cm male	50 ft., 3/8" male	air hose	to deliver air to drill	Trident Diving Equipment	each	$177.02	$177.02	1	$177.02		
	Cable cutter	25.4 cm	10 in		fragment elkhorn	Pittsburgh	each	$6.99	$6.99	5	$34.95		
	Wet tile saw	N/A	N/A	17.78 cm (7 in) blade diameter, 1 horse power	fragment staghorn coral	Q.E.P.	each	$98.00	$98.00	1	$98.00		
	Scissors & Folding Knife Set			stainless steel Dura Scissors	cut mono and lines	West Marine	each	$9.88	$9.88	4	$39.52	$733.98	
**Nursery Maintenance**	Wire Brush	18.4 cm	7-1/4 in	stainless-steel plastic handled	clean nursery	Gordon Brush Mfg.	each	$1.67	$1.67	5	$8.35		$2,264.54
	Painter's tools	6.35 cm blade width	2.5 in blade width	steel	clean nursery	Home Depot	each	$6.47	$6.47	5	$32.35		
	Slip Joint Pliers	15.24 cm	6 in	forged steel, rust resistant	clean nursery	Home Depot	each	$3.49	$3.49	5	$17.45	$58.15	

1
