# Supplementary material for: Acropora cervicornis and Acropora palmata cultured on a low maintenance line nursery design in The Bahamas
Source: PLoS One. 2022 Apr 25;17(4):e0267034. doi: 10.1371/journal.pone.0267034 (PMC9037939; doi:10.1371/journal.pone.0267034)
Supplement: S2 Table — Peripheral activities, such as travel time to and from sites and preparation of activities, are not included. Costs are in USD ($). (PDF) [file pone.0267034.s002.pdf]

| | | *Specifications* | *Laborers (# of people)* | *Labor (hours)* | *Cost (US $) for One Nursery* |
|---|---|---|---|---|---|
| **Construction of Nursery** | Fixed Costs (equipment needed to construct nursery) | Pneumatic power tools and accessories, crimpers, etc | | | $ 733.98 |
| **Labor** | Day 1 | Construction of vertical and horizontal lines | 2 | 2 | $34.60 |
| | | Coral cutting and stringing (n=128) | 4 | 12 | $415.20 |
| | | Drilling anchors | 2 | 1 | $17.30 |
| | Day 2 | Horizontal and vertical line Placement | 3 | 1.5 | $38.93 |
| | | Coral Placement on horizontal lines | 3 | 1.5 | $38.93 |
| **Materials** | SCUBA gear | 6 sets | | | |
| | SCUBA tanks | 8 | | | |
| | Vertical line | 3 custom made lines | | | $ 285.00 |
| | Buoys | 3 | | | $ 353.97 |
| | Underwater epoxy | 1 set to secure vertical lines | | | $ 97.74 |
| | Hardware | Shackles, chains, eyebolts, crimps, long line clips | | | $ 693.43 |
| | Monofilament | Lines | | | $ 42.27 |
| | | **Subtotal** | | **18** | **$ 2,017.37** |
| **Nursery Maintenance** | | | | | |
| **Labor** | Two visits per year | Cleaning and repairing lines | 5 | 20 | **$865** |
| **Materials** | SCUBA gear | 5 sets | | | |
| | SCUBA tanks | 20 | | | |
| | Cleaning tools | Wire brush, pliers and painters tool (5 sets) | | | **$ 58.15** |
| | | **Total** | | **38** | **$ 2,940.52** |
| | | **Initial cost per coral individual** | | | **$ 22.97** |

1 Labor costs were based off of minimal wage in 2018 ($8.65 US).
