# Supplementary figures and images for: Acropora cervicornis and Acropora palmata cultured on a low maintenance line nursery design in The Bahamas
Source: PLoS One. 2022 Apr 25;17(4):e0267034. doi: 10.1371/journal.pone.0267034 (PMC9037939; doi:10.1371/journal.pone.0267034)

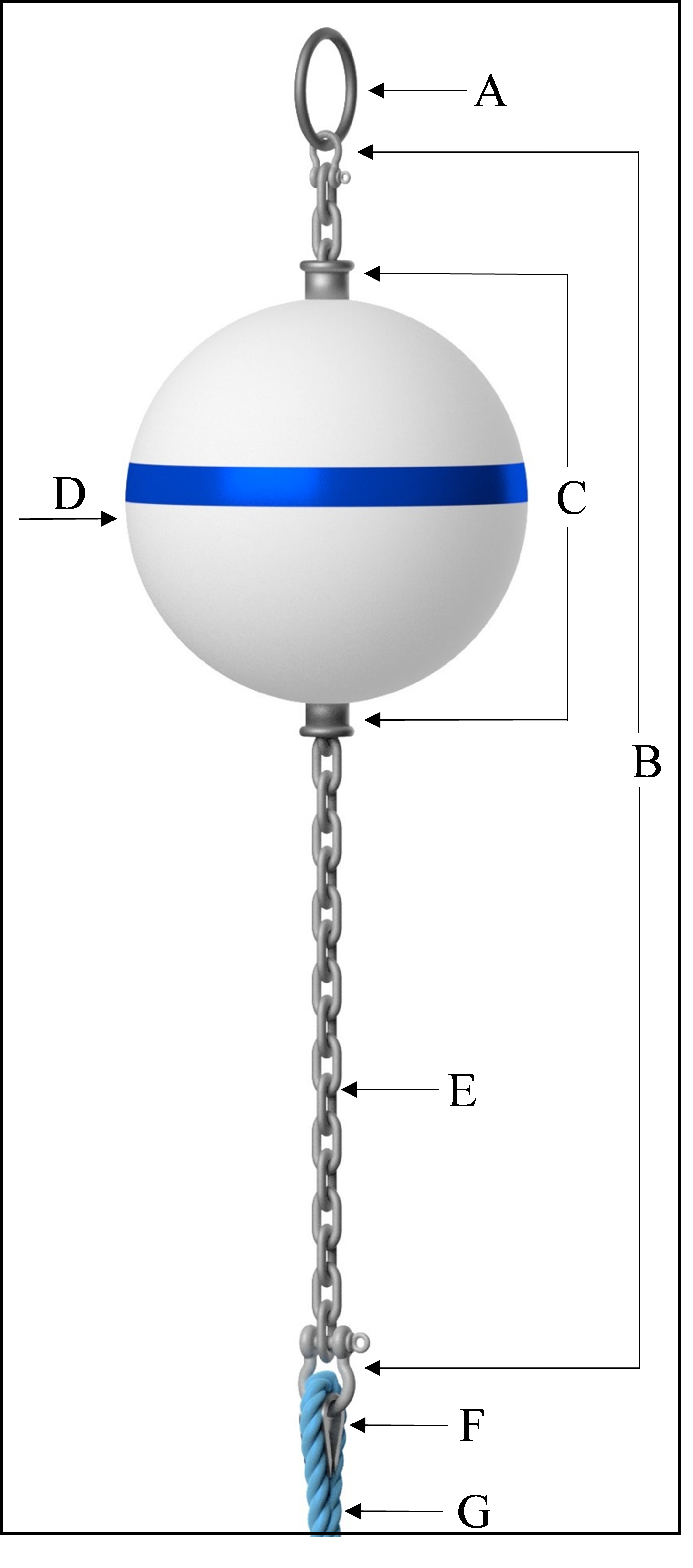

Supplement: S1 Fig — There are three vertical lines at which the top consisted of: (A) o-ring (B) 8 mm (5/16) anchor shackle (C) buoy collars (D) mooring buoy (E) steel chain (F) thimble (G) custom-ordered vertical line. (TIF) [file pone.0267034.s003.tif]

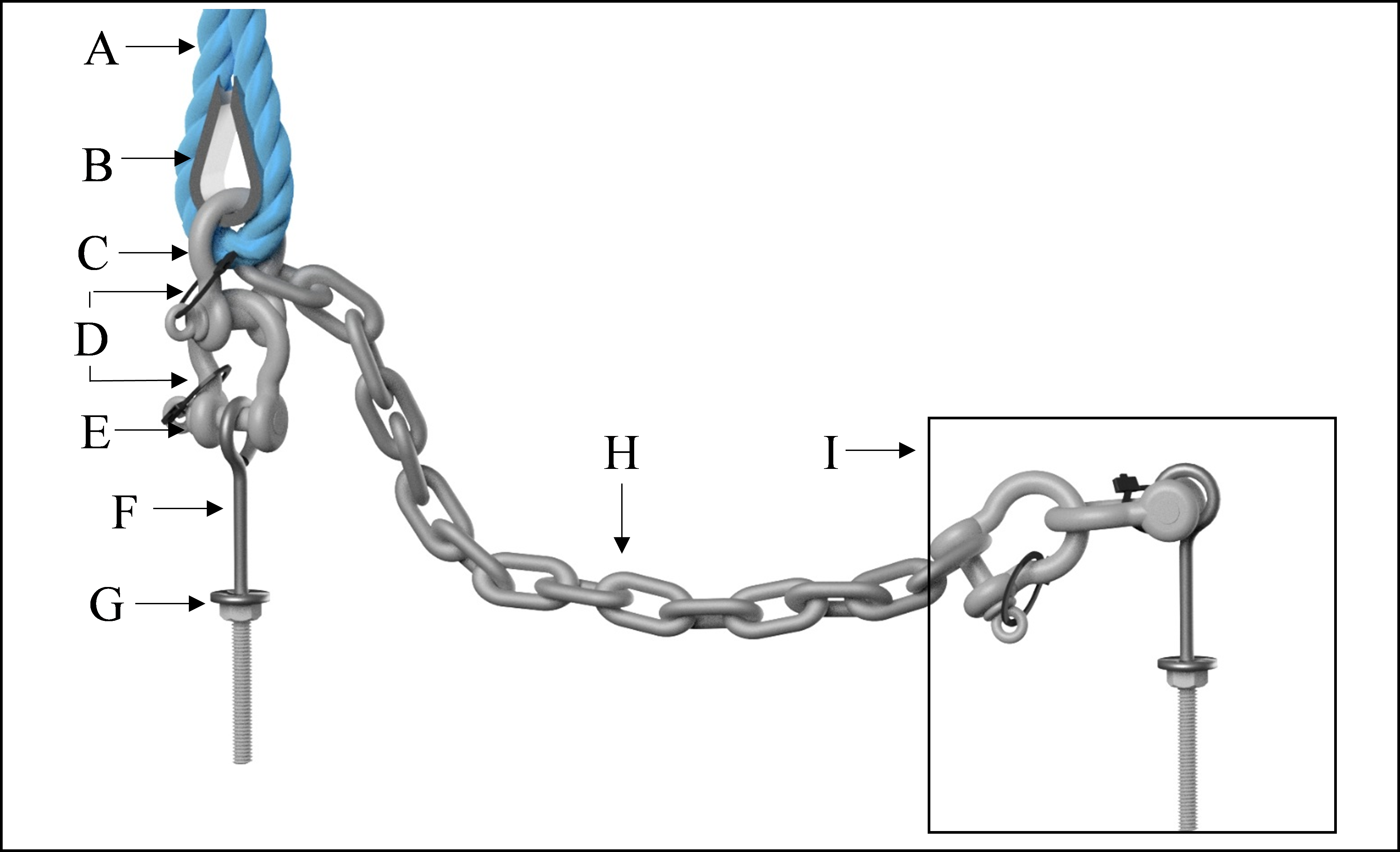

Supplement: S2 Fig — (A) custom-ordered vertical line (B) thimble (C) 13 mm (½ in) anchor shackle (D) cable ties (E) 8 mm (5/16 in) anchor shackle (F) eyebolt (G) nut and washer (H) 13 mm (1/2 in) steel chain (I) second eyebolt set. (TIF) [file pone.0267034.s004.tif]

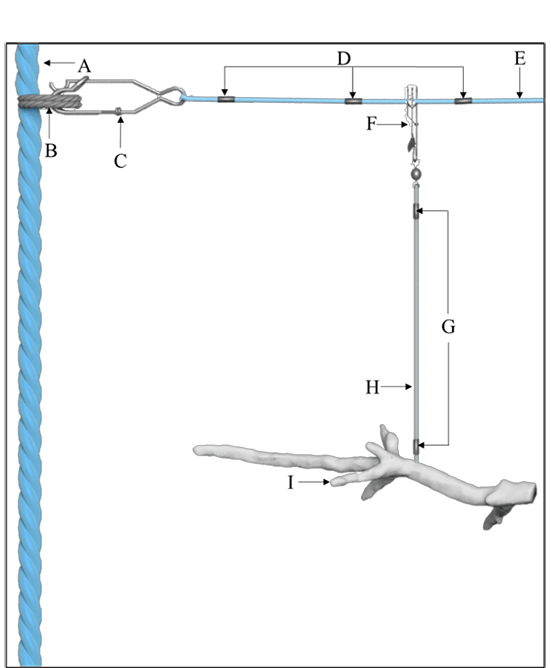

Supplement: S3 Fig — (A) custom-ordered vertical line (B) Two loops of 0.47 cm (3/16 in) diameter nylon line (C) 12.7 cm (5 in) stainless steel long line clip (D) 3.0 mm double barrel crimp (E) 2.8 mm diameter monofilament (F) 7.62 cm (3 in) long line clip (G) 2.0 mm double barrel crimps (H) 1.8 mm diameter monofilament line and (I) coral input. (TIF) [file pone.0267034.s005.tif]
